# ALT Donor Area Compartment Syndrome: A Rare and Devastating Complication

**DOI:** 10.1055/s-0044-1795149

**Published:** 2024-12-31

**Authors:** Gloria Alsina, Teresa Nunez-Villaveirán, Daniel Camporro, Clara Martin

**Affiliations:** 1 Plastic Surgery Unit, Mutua Sanitaria Mutual MC, Barcelona, Spain; 2 Plastic Surgery Unit, Hospital General de Granollers, Barcelona, Spain; 3 Department of Plastic Surgery, Hospital Universitario Central de Asturias, Spain

**Keywords:** compartment syndrome, ALT flap, microsurgery, quadriceps reconstruction

## Abstract

We present two cases of acute compartment syndrome (ACS) in the donor area of an anterolateral thigh (ALT) flap, with extensive necrosis of the quadriceps muscle. An innervated latissimus dorsi (LD) flap was used to cover the defect and restore functionality of the lower extremity. ACS in the ALT donor area is extraordinarily uncommon. Its mechanism seems to be multifactorial, being more frequent in young athletic males and, possibly, in patients with anatomical vascular variations. It is important to detect it promptly to prevent irreversible muscular ischemia. Intense pain is an alarm sign but might be absent in patients with epidural infusion pumps. We recommend caution with the use of this analgesia and frequent monitoring of the donor area during the early postoperative period. The free functional LD muscle flap is an option to treat full-thickness quadriceps damage, allowing for wound closure, soft-tissue coverage, and restoration of the knee extension movement.

## Introduction

The anterolateral thigh (ALT) flap usually has a low donor site morbidity. 1 We present two cases of severe acute compartment syndrome (ACS) in its donor area.

## Case Reports

### Case 1

A 37-year-old woman with a body mass index (BMI) of 32.5 presented with tibial pseudoarthrosis and sequelae of skin necrosis 1 year after left tibial and fibular fractures. The fracture focus was debrided, a bone graft was fixed, and the skin defect was covered with a 21 × 7 cm right fasciocutaneous ALT flap based on a myocutaneous perforator with a 5-cm intramuscular course, harvesting and closing the donor area within 105 minutes. The donor area skin was closed primarily under no apparent tension, without closing the muscle fascia, with a suction drain, and a noncircular dressing. The patient was taken to the postanesthetic care unit (PACU) with an epidural analgesia steady infusion pump.

**Fig. 1:**
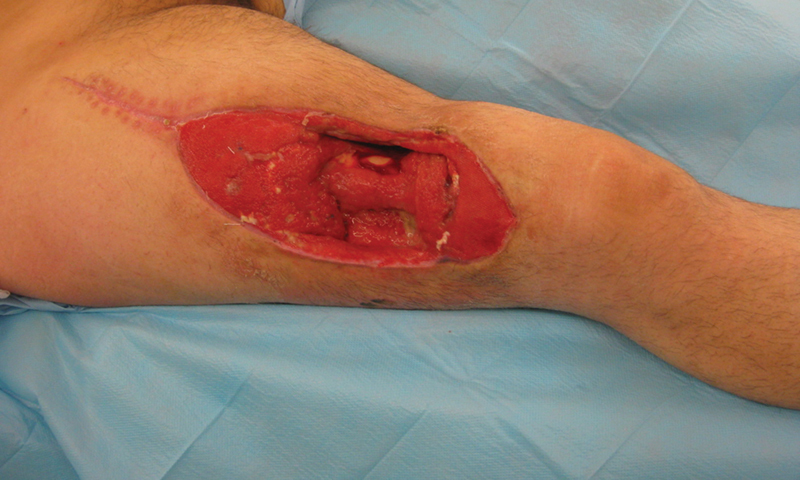
Thigh defect at the third postoperative week.

On postoperative day (POD) 1, the right thigh was under tension and, after removing the epidural catheter, the patient complained of intense pain. The wound was opened showing an edematous quadriceps with grayish-colored fascicles. It was left open with a vessel-loop shoelace technique, without further pain but no muscle improvement. On POD4, full-thickness necrosis of the quadriceps was debrided in the operating room (OR) to the periosteal plane of the femur, leaving a 15-cm-long defect ( Fig. 1 ).

**Fig. 2:**
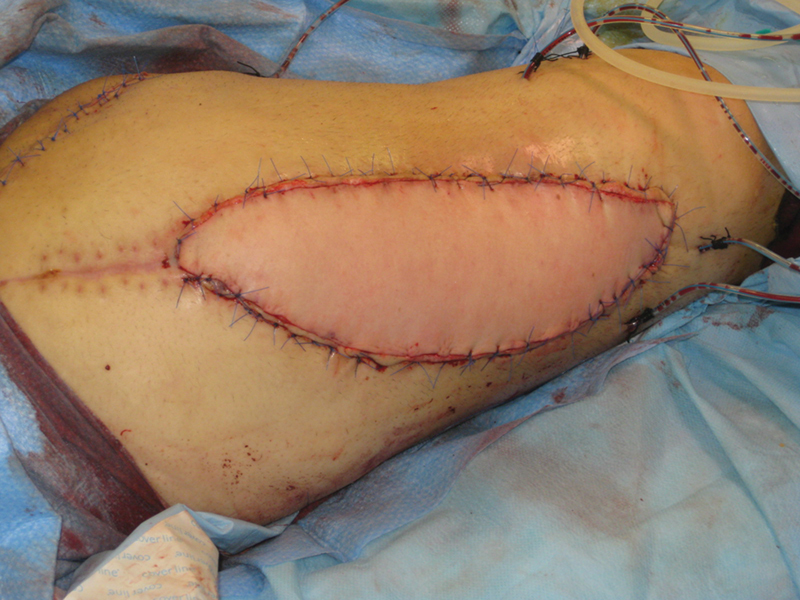
Immediate postoperative result after reconstruction with a free functional latissimus dorsi flap.

At postoperative month (POM) 1, a musculocutaneous latissimus dorsi (LD) flap was used to cover the quadriceps defect. Anastomoses were performed end to side to the femoral artery and end to end to the saphenous vein. The thoracodorsal nerve was anastomosed to a branch for the rectus femoris muscle ( Fig. 2 ).

**Fig. 3:**
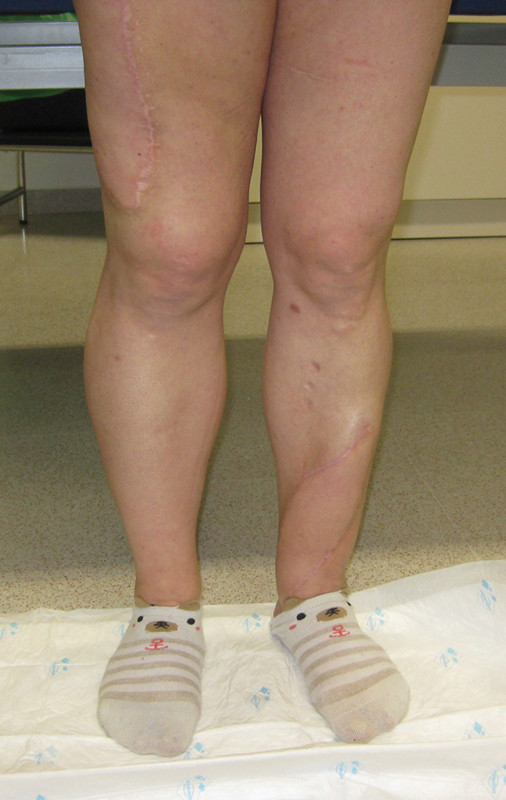
Results at postoperative month 14. The patient is able to extend her knee 45 degrees, and walk and climb stairs without the aid of a crutch.

At POM 10, an electromyogram (EMG) showed motor unit potentials in the LD flap. At POM 14, she was able to contract the LD flap extending her knee 45 degrees. She is now able to walk progressively and climb stairs without the aid of a crutch ( Fig. 3 ).

### Case 2

**Fig. 4:**
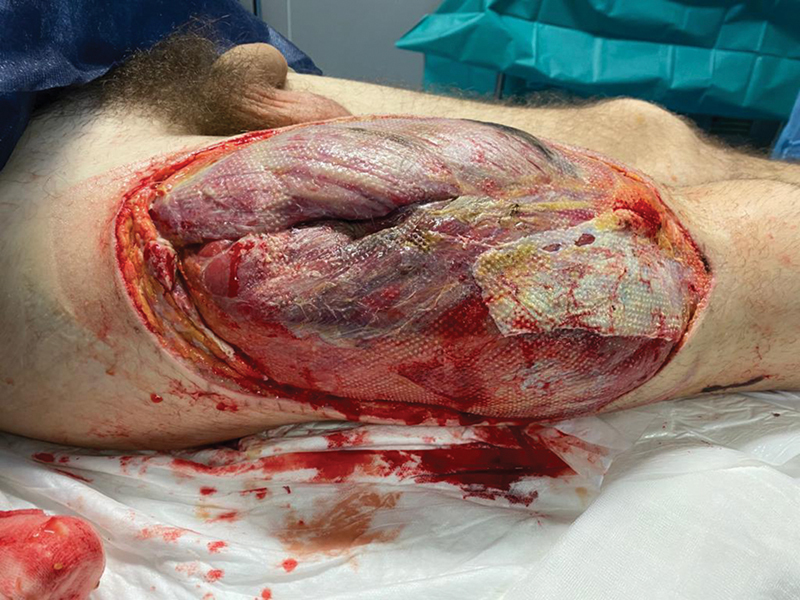
Postoperative day 1 after opening the thigh wound with intense edema and damage to the quadriceps muscle.

**Fig. 5:**
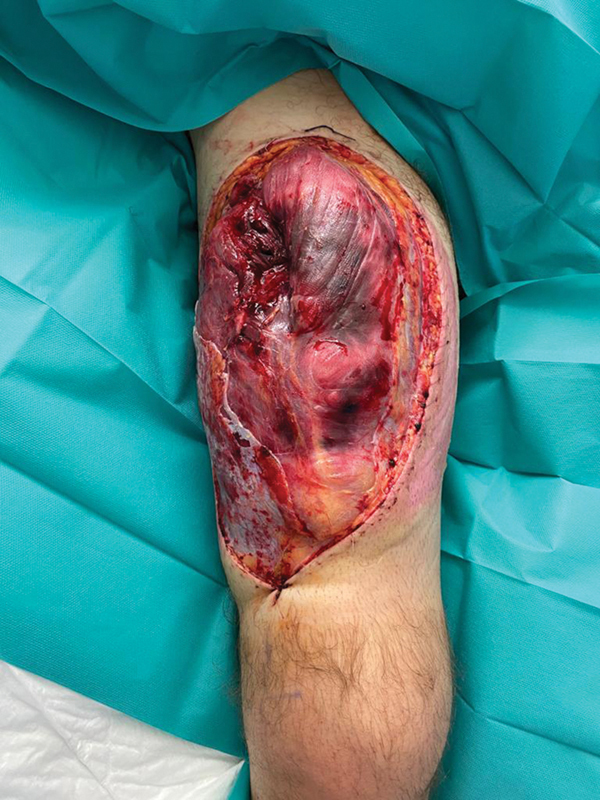
Thigh defect after debridement at postoperative week 1.

**Fig. 6:**
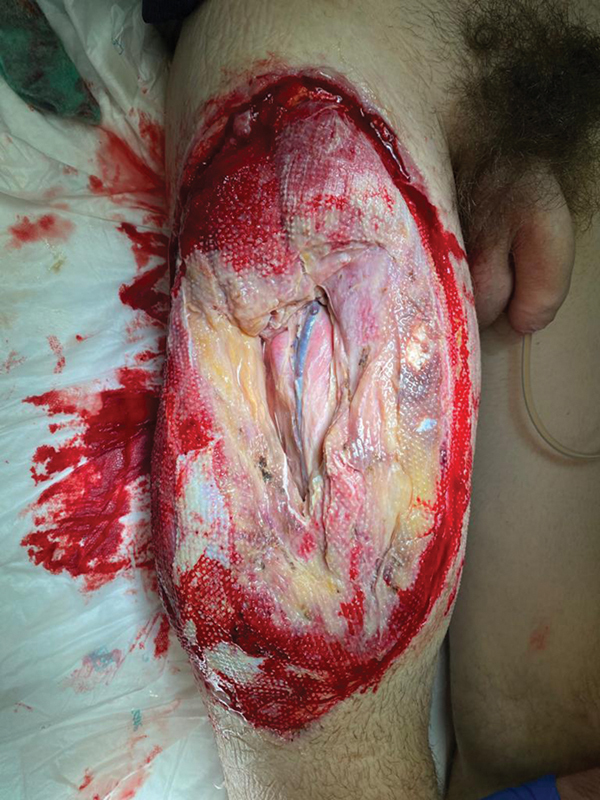
Thigh defect at postoperative week 2.

A 24-year-old man presented with necrosis of a part of the left sole and toes, Lisfranc and Chopart fracture-dislocations, and multiple phalanx fractures, after a crush injury with a forklift. After amputation of the toes and necrotic tissue debridement, the defect was covered with a contralateral 9 × 18 cm ALT free flap based on a myocutaneous perforator, harvesting and closing the donor area within 55 minutes. His thigh girth measured 40 cm. The donor area skin was closed primarily under slight tension, without closing the muscle fascia, without a suction drain, and a noncircular dressing. The patient was taken to the PACU with an epidural analgesia steady infusion pump. On POD1, the right lower extremity was edematous and cold with no peripheral pulses. The ALT donor area wound was opened, and pressure was taken from all lower limb compartments (30 mm Hg in the internal thigh and 43, 40, and 30 mm Hg in the anterior, external, and posterior leg compartments, respectively). Fasciotomies of the internal thigh and all leg compartments were performed, and a wound VAC was applied. There was full-thickness necrosis of the quadriceps, gracilis, peroneus brevis and longus, and tibialis anterior muscles ( Figs. 4 , 5 , 6 and , 7 ). Wound VAC reservoir changes were required twice a day during the first week, and four times a day the next 2 weeks due to extensive edema. On POD21, an EMG showed injury of the femoral nerve and sciatic nerve, denervation of their corresponding muscles, and sensory loss in all the sciatic territory. On POD22, fasciotomies were closed after partial resection of the tibialis anterior, gracilis, and peroneus muscles ( Fig. 5 ). On POD27, the femoral defect was covered with an LD flap with end-to-end anastomoses to epigastric vessels, but it was lost due to an artery thrombus. A contralateral LD flap was raised and anastomosed end to side to the femoral artery and end to end to a femoral vein satellite branch. The thoracodorsal nerve was anastomosed to a branch of the femoral nerve ( Fig. 6 ). The patient underwent neurolysis of the tibial and common fibular nerves due to neuropathic pain at POM6. At POM18, he was able to walk without crutches or braces. LD flap contraction allowed for knee stability but not for extension. EMG showed recovery of plantar sensation and partial recovery of the sciatic and femoral nerves.

## Discussion

ACS occurs when tissue pressure increases within a closed space, exceeding the capillary perfusion pressure. 2 Extrinsic cause factors include wide flaps, poor skin laxity, thick subcutaneous fat, and tight circumferential dressings. Intrinsic factors include hematomas, postoperative swelling, muscle volume, and tone. 2 3 4

The widths of our ALT flap skin paddles were 7 and 9 cm, and the donor area was closed primarily without excessive tension, not closing the fasciotomy. These widths usually do not require skin grafts for closure. However, based on our experience we now recommend a low threshold for closing donor sites with a split-thickness skin graft when the wound tension is high, irrespective of the width of the flap. This is particularly important in obese patients, who have a thicker subcutaneous fat, and in the younger, athletic patients, who have an increased risk of ACS, possibly due to their increased muscle volume and tone, 2 3 and skin firmness. 5 6 Skin grafts placed over bare muscle in the thigh do not cause any functional deficit, while failure to place skin grafts when required may lead to functional complications, as in our two patients. Furthermore, often this graft can safely be resected after the period of edema has passed to improve the cosmesis. 7 None of our patients had coagulation disorders, hematomas, or circumferential dressings. Only one of our patients was obese.

Significant swelling may develop when flap elevation without pedicle division is completed hours before the inset, or with long ischemic times (above 2 hours) with pneumatic tourniquets. 5 Postoperative swelling may also increase in complex vascular pedicle intramuscular dissections, or if there is muscle ischemia, thrombosis, or venous obstruction. 2 3 4 8 However, both flaps were harvested and closed completed promptly, with no pneumatic tourniquets, and immediately inset into the defects.

Pain, usually the earliest sign of ACS, was absent because of the use of epidural pump analgesia, delaying our patients' diagnosis. Thus, we stopped using postoperative epidural analgesia and performed the first dressing change of the donor site on POD1. Our other postoperative protocol includes initial bed rest with elevation of the leg, prophylactic heparin and 100 mg aspirin, in vivo optical spectroscopy monitoring of the flap, and daily donor area dressing changes. Other preventive methods could include incisional negative pressure dressings, but they should not be a substitute for skin grafting.

Partial necrosis of the rectus femoris muscle has been described only twice after ALT harvest, possibly associated with vascular anatomic variations in which the ALT pedicle is the dominant pedicle of the rectus femoris muscle. 1 9 However, we always maintain and verify the viability of the rectus femoris after raising an ALT flap and dissect the pedicle distal to the rectus femoris pedicle.

ACS in the donor area of an ALT flap has been documented in the literature in only four other cases. 2 3 10 Similarly to them, the causes of ACS were probably multifactorial. Our hypothesis is that, despite resecting the deep fascia with the flap, the tight closure of the overlying skin created an additional constricting layer around the muscle compartment. Postoperative swelling from the dissection of intramuscular perforators led to increased intracompartmental pressure. The use of epidural analgesia masked the pain, and the increased intracompartmental pressure resulted in muscle ischemia, which in turn increased capillary permeability and intramuscular edema, creating a vicious cycle leading to ACS.

Free functional myocutaneous LD flaps anastomosed to a branch of the femoral nerve were used in both patients, achieving wound and soft tissue closure, and restoration of quadriceps femoris joint movement. 11 The LD is the largest available strap muscle for transfer with suitable fiber length for the required range of contraction, 12 necessary viable bulk, a suitable range of motion, and adaptable shape for the recipient area. 11 Its motor innervation comes from a single motor (thoracodorsal) nerve traveling with the vascular (subscapular) pedicle with a length that facilitates tension-free microanastomoses. 13

**Fig. 7:**
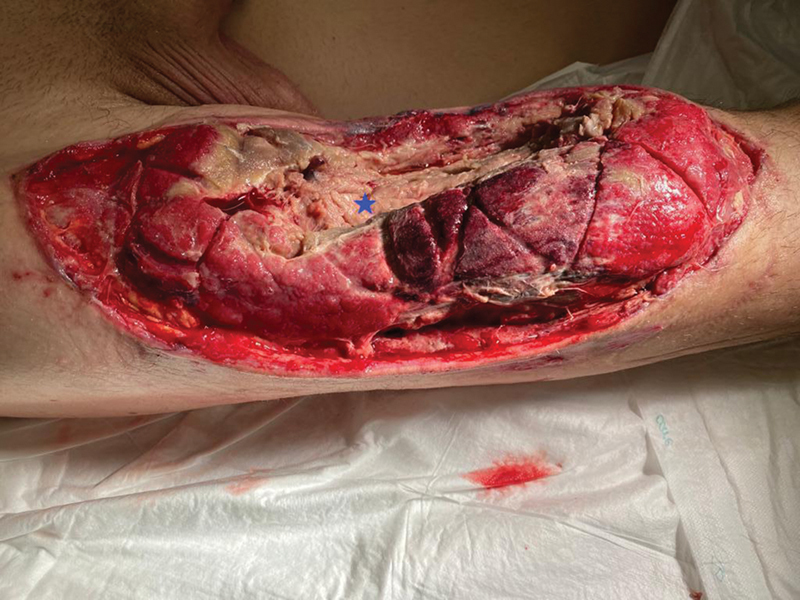
Thigh defect after third debridement at postoperative week 3, with full-thickness necrosis of the quadriceps, gracilis, peroneus brevis, and longus and tibialis anterior muscles. The *blue star* corresponds to the femur and remnants of the vastus intermedius over it.

## References

[1] Wong C H Wei F C Anterolateral thigh flap Head Neck 2010 32 04 529 540 19672962 10.1002/hed.21204

[2] Addison P D Lannon D Neligan P C Compartment syndrome after closure of the anterolateral thigh flap donor site: a report of two cases Ann Plast Surg 2008 60 06 635 638 18520198 10.1097/SAP.0b013e3181453b7a

[3] Moon B M Pae W S Compartment syndrome as a donor-site complication of anterolateral thigh free flap: a case report Arch Hand Microsurg. 2022 27 01 83 87

[4] Hwang J H Kim K S Lee S Y A case of nonisland pedicled foot fillet flap for below-knee amputation stump wound: treatment option for compartment syndrome after fibular free flap surgery J Korean Med Sci 2014 29 02 305 308 24550664 10.3346/jkms.2014.29.2.305 PMC3924016

[5] Shindo M Fong B P Funk G F Karnell L H The fibula osteocutaneous flap in head and neck reconstruction: a critical evaluation of donor site morbidity Arch Otolaryngol Head Neck Surg 2000 126 12 1467 1472 11115284 10.1001/archotol.126.12.1467

[6] Klein S Hage J J Woerdeman L A Donor-site necrosis following fibula free-flap transplantation: a report of three cases Microsurgery 2005 25 07 538 542, discussion 542 16184523 10.1002/micr.20169

[7] Saleem M Hashim F Babu Manohar M Compartment syndrome in a free fibula osteocutaneous flap donor site Br J Plast Surg 1998 51 05 405 407 9771370 10.1054/bjps.1998.0005

[8] Sun G Yang X Wen J Wang A Hu Q Tang E Treatment of compartment syndrome in donor site of free fibula flap after mandibular reconstruction surgery Oral Surg Oral Med Oral Pathol Oral Radiol Endod 2009 108 05 e15 e18 19836708 10.1016/j.tripleo.2009.07.001

[9] Maruccia M Di Taranto G Nicoli F Ciudad P Giudice G Chen H C Rectus femoris muscle necrosis: an underrated donor-site complication of free anterolateral thigh flap J Plast Reconstr Aesthet Surg 2017 70 07 972 974 28410985 10.1016/j.bjps.2017.03.007

[10] Qu W Pan J Jin H Wang X Tian H Acute compartment syndrome secondary to anterolateral thigh flap harvesting in a pediatric patient: a case report Medicine (Baltimore) 2020 99 28 e21216 32664172 10.1097/MD.0000000000021216 PMC7360265

[11] Muramatsu K Ihara K Miyoshi T Yoshida K Hashimoto T Taguchi T Transfer of latissimus dorsi muscle for the functional reconstruction of quadriceps femoris muscle following oncological resection of sarcoma in the thigh J Plast Reconstr Aesthet Surg 2011 64 08 1068 1074 21474401 10.1016/j.bjps.2011.03.010

[12] Hallock G G Restoration of quadriceps femoris function with a dynamic microsurgical free latissimus dorsi muscle transfer Ann Plast Surg 2004 52 01 89 92 14676706 10.1097/01.SAP.0000070433.06200.2C

[13] Taylor G I Gianoutsos M P Morris S F The neurovascular territories of the skin and muscles: anatomic study and clinical implications Plast Reconstr Surg 1994 94 01 1 36 8016221 10.1097/00006534-199407000-00001

